# Relationship among bats, parasitic bat flies, and associated pathogens in Korea

**DOI:** 10.1186/s13071-021-05016-6

**Published:** 2021-09-27

**Authors:** Haeseung Lee, Min-Goo Seo, Seung-Hun Lee, Jae-Ku Oem, Seon-Hee Kim, Hyesung Jeong, Yongkwan Kim, Weon-Hwa Jheong, Oh-Deog Kwon, Dongmi Kwak

**Affiliations:** 1 College of Veterinary Medicine, Kyungpook National University, 80 Daehak-ro, Buk-gu, Daegu, 41566 South Korea; 2 Veterinary Drugs and Biologics Division, Animal and Plant Quarantine Agency, 177 Hyeoksin 8-ro, Gimcheon, Gyeongbuk 39660 South Korea; 3 College of Veterinary Medicine, Chungbuk National University, 1 Chungdae-ro, Seowon-gu, Cheongju, Chungbuk 28644 South Korea; 4 College of Veterinary Medicine, Jeonbuk National University, 79 Gobong-ro, Iksan, Jeonbuk 54596 South Korea; 5 National Institute of Wildlife Disease Control and Prevention, 1 Songam-gil, Gwangsan-gu, Gwangju, 62407 South Korea

**Keywords:** Bat, Bat fly, Blood-borne pathogen, Phylogeny, Prevalence

## Abstract

**Background:**

Bats are hosts for many ectoparasites and act as reservoirs for several infectious agents, some of which exhibit zoonotic potential. Here, species of bats and bat flies were identified and screened for microorganisms that could be mediated by bat flies.

**Methods:**

Bat species were identified on the basis of their morphological characteristics. Bat flies associated with bat species were initially morphologically identified and further identified at the genus level by analyzing the cytochrome c oxidase subunit I gene. Different vector-borne pathogens and endosymbionts were screened using PCR to assess all possible relationships among bats, parasitic bat flies, and their associated organisms.

**Results:**

Seventy-four bat flies were collected from 198 bats; 66 of these belonged to Nycteribiidae and eight to Streblidae families. All Streblidae bat flies were hosted by *Rhinolophus ferrumequinum*, known as the most common Korean bat. Among the 74 tested bat flies, PCR and nucleotide sequencing data showed that 35 (47.3%) and 20 (27.0%) carried *Wolbachia* and *Bartonella* bacteria, respectively, whereas tests for *Anaplasma*, *Borrelia*, *Hepatozoon*, *Babesia*, *Theileria*, and *Coxiella* were negative. Phylogenetic analysis revealed that *Wolbachia* endosymbionts belonged to two different supergroups, A and F. One sequence of *Bartonella* was identical to that of *Bartonella* isolated from Taiwanese bats.

**Conclusions:**

The vectorial role of bat flies should be checked by testing the same pathogen and bacterial organisms by collecting blood from host bats. This study is of great interest in the fields of disease ecology and public health owing to the bats’ potential to transmit pathogens to humans and/or livestock.

**Graphical abstract:**

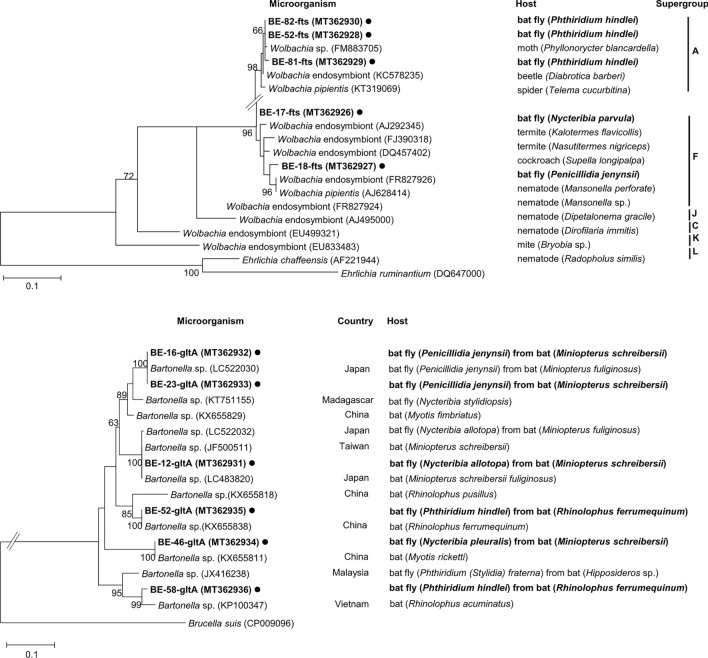

**Supplementary Information:**

The online version contains supplementary material available at 10.1186/s13071-021-05016-6.

## Background

As a group, bats include approximately 1432 species [1]. Several bat species are key to their ecosystems and also act as pathogen reservoirs [2]. Bat viruses are of great interest in disease ecology and public health owing to their potential to infect humans and livestock [3]. Moreover, bacteria and protozoa have also been detected in bats. *Bartonella* bacteria and *Trypanosoma cruzi* protozoa, which are associated with bats, have also been detected in humans, making these bat-related organisms an urgent public health concern [4, 5]. Bats harbor several ectoparasites, including bat flies, fleas, and certain arachnids, such as mites and ticks. Bat fly families Nycteribiidae and Streblidae belong to the superfamily Hippoboscoidea, which also includes the families Hippoboscidae (louse flies and keds) and Glossinidae (tsetse flies), and are the most common bat ectoparasites [2, 6].

Currently, 275 species of 21 genera of nycteribiids and 227 species of 31 genera of streblids have been described [7]. The importance of louse flies as a potential vector has been well studied. Recently, it has been confirmed that *Anaplasma ovis*, *Bartonella* spp., *Rickettsia* spp., and *Trypanosoma* spp. are present in these insects [8–10]. Bat flies are also considered vectors. In recent studies, bat ectoparasite burden was found to be proportional to *Bartonella* infection; moreover, *Bartonella* spp. were also detected in bat flies and host bats, underscoring the parasite vector potential. However, more research is warranted [11, 12]. Furthermore, it has not been demonstrated that bat flies transmit *Bartonella* bacteria. The vector potential of bat flies was demonstrated only in *Polychromophilus* spp. [13].

Bat flies are obligate ectoparasites for bats and include endosymbiotic prokaryotes that are not yet well understood; however, it is assumed that they establish a symbiotic relationship with mutualistic bacteria [14]. Members of the superfamily Hippoboscoidea require milk secretion for larval development, and certain bacteria such as *Bartonella* and *Wolbachia* can be vertically transmitted during this process. These bacteria can also be horizontally transmitted through parasitoids or via contact with contaminated saliva [15, 16]. However, horizontal transmission has not been proven in bat flies or any other hippoboscids.

*Wolbachia* is a bacterium belonging to the order Rickettsiales, which includes the genera *Anaplasma*, *Ehrlichia*, and *Rickettsia*. *Wolbachia* influences host reproduction through extensive symbiotic interactions in some species and is estimated to be present in up to 66% of insect species [17]. *Wolbachia* has become an integral component of vector-mediated disease control due to its ability to spread through insect populations and influence vector competence through pathogen protection [18]. *Bartonella* spp. are parasitic bacteria that infect the red blood cells of vertebrates. Several different bacterial species [19], including *Bartonella mayotimonensis*, are associated with bats, some of which are zoonotic and can cause disease in humans [5].

However, to date, few studies have examined the pathogenic relationships among bat flies, although previous studies reported the possibility of *Bartonella* and *Wolbachia* bacteria occurring transiently [20, 21]. In general, the high degree of host specificity in bat flies reduces the likelihood of interspecies transmission of pathogens, but bat flies are still likely to carry transmissible pathogens within the host population [7]. Most previous studies on microorganisms, including those on *Wolbachia* spp., in bat flies have focused on endosymbiotic characteristics or distribution [2, 20].

In Korea, many vector-mediated diseases and causative agents, including *Anaplasma*, *Bartonella*, and *Borrelia*, occur in humans [22–24]. However, there are few data regarding bats and bat flies in Korea. Furthermore, a recent Korea-focused study assessed the local distribution of bat flies without considering the pathogens mediated by these flies [25]. Therefore, the purpose of this study was to investigate the relationships among bats, parasitic bat flies, and their associated bacteria in Korea.

## Methods

### Study area, sample collection, and species identification of bats and bat flies

**Fig. 1:**
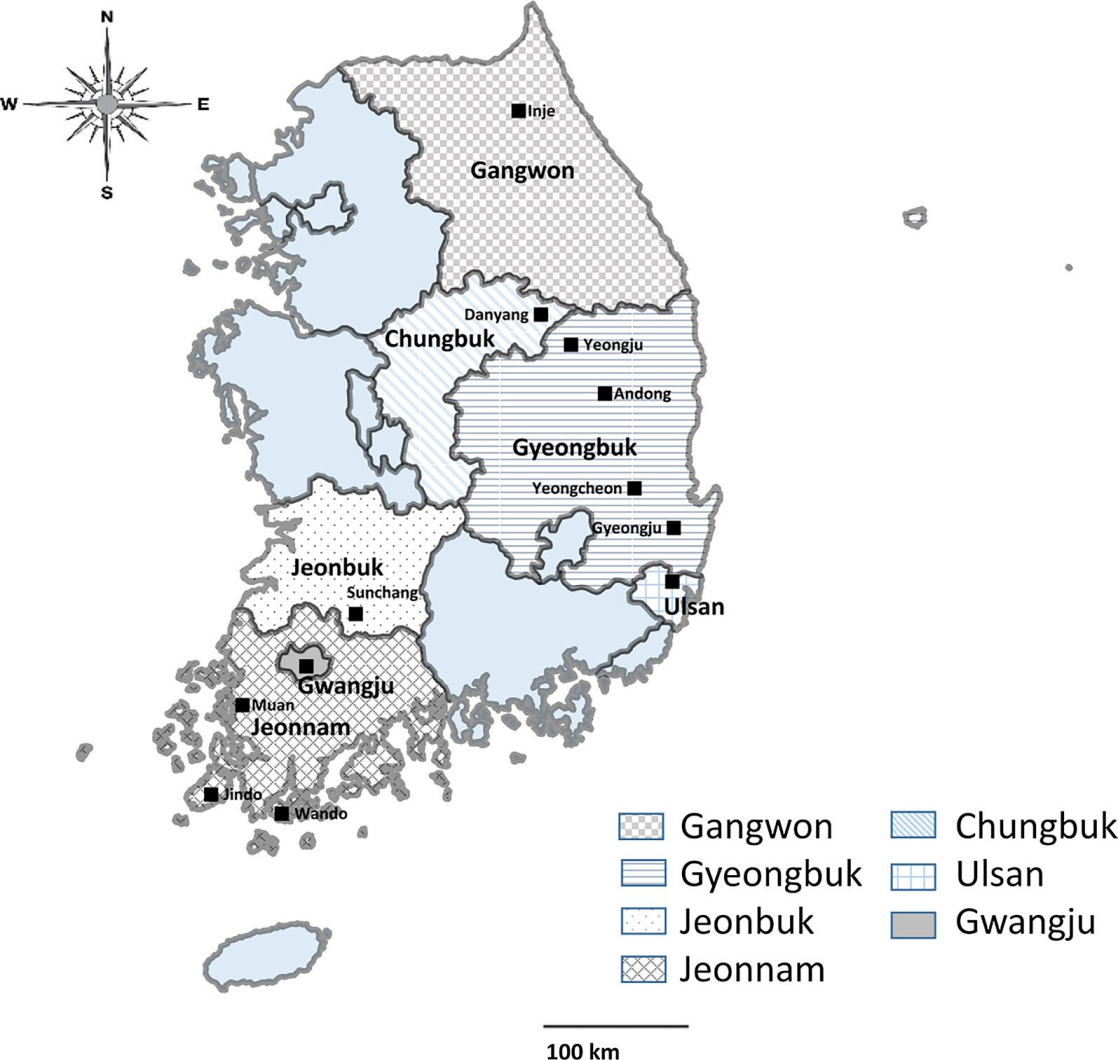
Map of South Korea. Bats and bat flies were collected from regions marked with squares

Bats and bat flies were collected from caves, forests, and abandoned mines in 12 cities across seven provinces [Gangwon (Inje), Chungbuk (Danyang), Gyeongbuk (Yeongju, Andong, Yeongcheon, and Gyeongju), Ulsan, Jeonbuk (Sunchang), Gwangju, and Jeonnam (Muan, Jindo, and Wando)] of Korea from February 2016 to August 2017 (Fig. 1). A total of 198 dead bats were found, and 74 bat flies were collected and immersed in 70% ethyl alcohol solution. The bat flies were collected by ecologists licensed from the National Institute of Environmental Research, Korea. The bat species were identified based on their morphological characteristics as previously described [26, 27], and all collection-related information was provided from the ecologists.

**Fig. 2:**
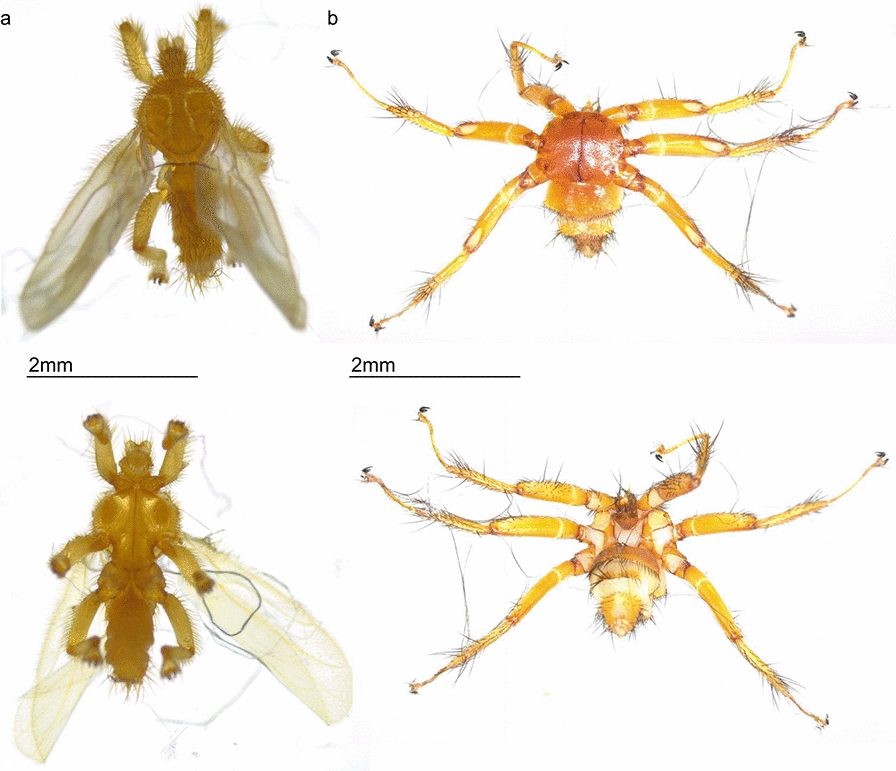
Typical morphology of bat files belonging to the family **a** Streblidae and **b** Nycteribiidae. Note the existence or absence of wings. Upper, dorsal view; lower, ventral view

Bat fly species were initially identified using key morphological characteristics, such as the presence or absence of wings, using a dissecting microscope (Fig. 2) [28]. The species were further identified at least at the genus level by analyzing the cytochrome c oxidase subunit I (*COI*) gene (approximately 658 bp length) [29, 30], which was amplified through PCR using invertebrate-specific primers [31, 32].

### DNA extraction and PCR assay

Bat fly DNA was extracted using the DNeasy® Blood & Tissue Kit (Qiagen, Hilden, Germany) as per the manufacturer’s instructions. An Infinite® 200 PRO NanoQuant (Tecan, Männedorf, Switzerland) plate reader was used to assess the quality and quantity of bat fly DNA by calculating the ratio of the absorbance at 260 nm and 280 nm. DNA samples were stored at − 20 °C until further use.

The commercially available AccuPower® HotStart PCR Premix kit (Bioneer, Daejon, Korea) was used for PCR. This premix product includes most of the elements required for PCR, including DNA polymerase, dNTPs, reaction buffer, and metal ions, lyophilized in a single tube. For PCR amplification, primers, DNA template, and distilled water are added until the total volume of the mixture reaches 20 μl. Bat fly-mediated pathogens and bacterial organisms were detected by amplification using primers specific to a target gene in each microorganisms. All reactions were performed using 20 μl reaction mixture containing 16 μl distilled water, 1 μl of 10 μM of each primer pair, and 2 μl template DNA.

We amplified the 16S rRNA regions of Rickettsiales (*Anaplasma*, *Ehlrichia*, *Rickettsia*, and *Wolbachia* species) and *Coxiella* spp.; 5S–23S rRNA regions of *Borrelia* spp.; 18S rRNA regions of *Babesia*, *Theileria*, and *Hepatozoon* species; and internal transcribed spacer I (ITS-1) regions of *Bartonella* spp. [23, 33–36]. Positive DNA samples were confirmed using a second set of PCR primers to amplify other regions of the gene, including citrate synthase gene (*gltA*) of *Bartonella* spp. [37] and cell division protein FtsZ (*ftsZ*) of *Wolbachia* spp. [36]. These primers are listed in Additional file 1: Table S1, along with their expected amplicon sizes. All PCR amplifications were performed using the Mastercycler® nexus GSX1 (Eppendorf, Hamburg, Germany) under conditions outlined in Additional file 2: Table S2. The PCR products were electrophoresed on a 1% agarose gel stained with ethidium bromide. All amplicons were visualized and photographed using a UV transilluminator, and PCR-positive products were sent to Macrogen (Daejeon, Korea) for DNA sequencing analysis.

### Sequencing and phylogenetic analysis

The obtained sequence data were aligned and edited using BioEdit 7.2.5 [38]. MEGA 7 was used to construct phylogenetic trees for each species using the maximum likelihood method with 1000 replicates based on the fragments of *COI*, *ftsZ*, and *gltA* of bat flies, *Wolbachia* spp., and *Bartonella* spp., respectively [39]. Reference sequence data were obtained using NCBI Web BLAST (http://www.ncbi.nlm.nih.gov/blast). The phylogenetic tree of *Wolbachia* spp. based on *ftsZ* was constructed using 27 GenBank database entries and *Ehrlichia* sp. as the outgroup. The phylogenetic tree of the *Bartonella* spp. based on *gltA* was constructed using 15 GenBank database entries and *Brucella* sp. as the outgroup.

## Results

### Identification of bat species

Overall, 11 species of seven genera belonging to three families were morphologically identified from 198 bats. One species each of Miniopteridae and Rhinolophidae and nine species of Vespertilionidae were found. The most common bat species was *Miniopterus fuliginosus* (32.8%, *n* = 65), followed by *Rhinolophus ferrumequinum* (29.3%, *n* = 58), *Myotis macrodactylus* (14.1%, *n* = 28), *Vespertilio sinensis* (7.1%, *n* = 14), *Myotis petax* (4.0%, *n* = 8), *Eptesicus serotinus* (3.5%, *n* = 7), *Myotis bombinus* (3.0%, *n* = 6), *Murina hilgendorfi* (2.5%, *n* = 5), *Hypsugo alaschanicus* (1.5%, *n* = 3), *Myotis ikonnikovi* (1.0%, *n* = 2), *Myotis aurascens* (0.5%, *n* = 1), and one unidentified specimen (Table 1).

**Table 1:** Bat distribution by location

	Bat species (*n*)	Chungbuk	Gangwon	Gwangju	Gyeongbuk	Jeonnam	Jeonbuk	Ulsan	Unknown	%
Miniopteridae (65)										
* Miniopterus*	*Miniopterus fuliginosus* (65)	16			7	1	39		2	32.8
Rhinolophidae (58)										
* Rhinolophus*	*Rhinolophus ferrumequinum* (58)	4		4	6	8	9	12	15	29.3
Vespertilionidae (74)										
* Eptesicus*	*Eptesicus serotinus* (7)			3	4					3.5
* Hypsugo*	*Hypsugo alaschanicus* (3)	2							1	1.5
* Murina*	*Murina hilgendorfi* (5)								5	2.5
* Myotis*	*Myotis aurascens* (1)	1								0.5
	*Myotis bombinus* (6)				6					3.0
	*Myotis ikonnikovi* (2)		2							1.0
	*Myotis macrodactylus* (28)	3				2	20		3	14.1
	*Myotis petax* (8)	6			2					4.0
* Vespertilio*	*Vespertilio sinensis* (14)	10							4	7.1
	Unidentified (1)				1					0.5
Total	(198)	42	2	7	26	11	68	12	30	100

### Identification of bat fly species

**Fig. 3:**
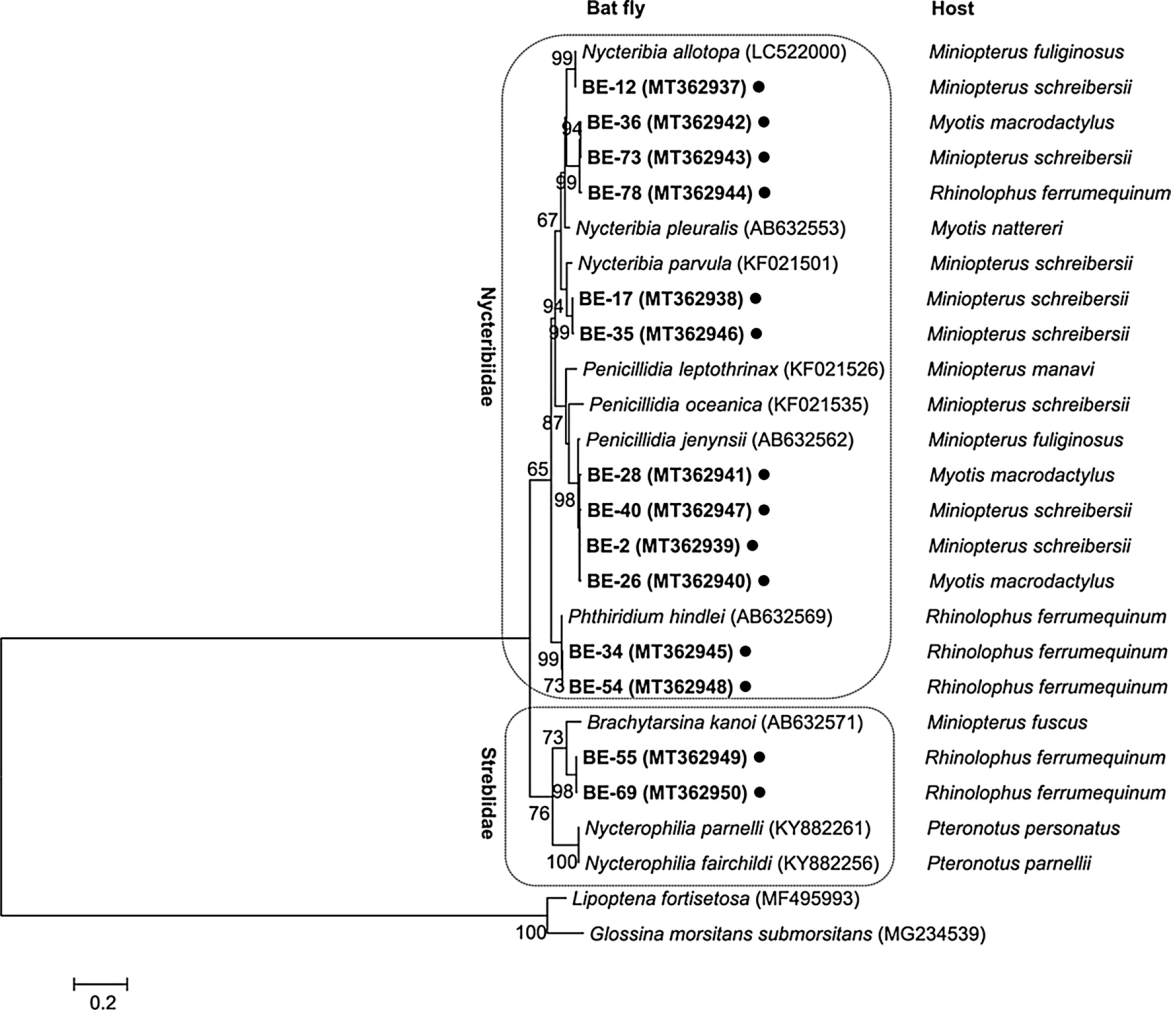
Phylogenetic tree based on bat fly cytochrome c oxidase subunit I gene-amplifying sequences. The ked and tsetse fly sequences were used as outgroups. Scale bar indicates an evolutionary distance of 0.20 nucleotides per position in the sequence. The black circles (●) indicate the bat fly sequences identified in this study

A total of 74 bat flies were collected from 198 bats. Ectoparasites other than bat flies were not detected. Organisms belonging to the families Nycteribiidae (89.2%, *n* = 66) and Streblidae (10.8%, *n* = 8) were identified in three species of host bat (Table 2). Among the Nycteribiidae specimens, five species from three genera were identified, and the % similarities of the bat fly species based on their GenBank match were as follows: *Nycteribia allotopa* (100% with LC522000), *Nycteribia parvula* (96.7% with KF021501), *Nycteribia pleuralis* (94.3% with AB632553), *Penicillidia jenynsii* (98.6% with AB632562), and *Phthiridium hindlei* (99.9% with AB632569). Among the Streblidae specimens, only one species of the genus *Brachytarsina* was identified, and % similarity of the bat fly species based on GenBank match was *Brachytarsina kanoi* (93.0% with AB632571) (Fig. 3; Table 2). Although it is the closest homology to this species, there is the probability of another closely related species apart from this. Most bat fly specimens were found on *M. fuliginosus* (51.4%, *n* = 38). All *P. hindlei* (*n* = 15) and *Brachytarsina* sp. (*n* = 8) were parasitic on the bat species *R. ferrumequinum*. Most *P. jenynsii* (family Nycteribiidae) flies were collected from the specimens obtained from the Jeonbuk Province (24/26), whereas only two such individuals were identified in specimens from the Chungbuk Province. *Brachytarsina* sp. (family Streblidae) was identified in three specimens from Gyeongbuk, three specimens from Jeonnam, and two specimens from Ulsan (Table 3).

**Table 2:** Collected host bat and bat fly species identification

Host bat species	No. of collected bat fly (%)								Total
	Nycteribiidae (66, 89.2%)							Streblidae (8, 10.8%)	
	*Nycteribia allotopa*	*Nycteribia parvula*	*Nycteribia* sp.^a^	*Nycteribia* sp.^b^	*Penicillidia jenynsii*	*Phthiridium hindlei*	Unidentified	*Brachytarsina* sp.^c^	
*Miniopterus fuliginosus*	2 (2.7)	2 (2.7)	2 (2.7)	2 (2.7)	22 (29.7)	0 (0.0)	8 (10.8)	0 (0.0)	38 (51.4)
*Myotis macrodactylus*	0 (0.0)	0 (0.0)	3 (4.1)	0 (0.0)	3 (4.1)	0 (0.0)	0 (0.0)	0 (0.0)	6 (8.1)
*Rhinolophus ferrumequinum*	0 (0.0)	0 (0.0)	1 (1.4)	0 (0.0)	1 (1.4)	15 (20.3)	5 (6.8)	8 (10.8)	30 (40.5)
Total	2 (2.7)	2 (2.7)	6 (8.1)	2 (2.7)	26 (35.1)	15 (20.3)	13 (17.6)	8 (10.8)	74 (100.0)

^a^The closest GenBank matching species is *Nycteribia pleuralis*

^b^The closest GenBank matching species is not identified

^c^The closest GenBank matching species is *Brachytarsina kanoi*

**Table 3:** Bat fly distribution by location^a^

Bat fly species	Bat host species (*n*)	Chungbuk	Gyeongbuk	Jeonbuk	Jeonnam	Ulsan	Unknown
Nycteribiidae (66)							
*Nycteribia allotopa* (2)	*Miniopterus fuliginosus* (2)	1		1			
*Nycteribia parvula* (2)	*Miniopterus fuliginosus* (2)	1		1			
*Nycteribia* sp.^b^ (6)	*Miniopterus fuliginosus* (2)	1		1			
	*Myotis macrodactylus* (3)	1		2			
	*Rhinolophus ferrumequinum* (1)			1			
*Nycteribia* sp.^c^ (2)	*Miniopterus fuliginosus* (2)			2			
*Penicillidia jenynsii* (26)	*Miniopterus fuliginosus* (22)	2		20			
	*Myotis macrodactylus* (3)			3			
	*Rhinolophus ferrumequinum* (1)			1			
*Phthiridium hindlei* (15)	*Rhinolophus ferrumequinum* (15)	3	3		3	5	1
Unidentified (13)	*Rhinolophus ferrumequinum* (5)			3	2		
	*Miniopterus fuliginosus* (8)	5		3			
Streblidae (8)							
*Brachytarsina* sp.^d^ (8)	*Rhinolophus ferrumequinum* (8)		3		3	2	
Total (74)		14	6	38	8	7	1

^a^Not detected in Gangwon and Gwangju regions

^b^The closest GenBank matching species is *Nycteribia pleuralis*

^c^The closest GenBank matching species is not identified

^d^The closest GenBank matching species is *Brachytarsina kanoi*

### Screening for pathogens and endosymbionts mediated by bat flies

Of the identified pathogens and endosymbionts, 35 specimens were part of the *Wolbachia* spp. (47.3%) and 20 specimens of the *Bartonella* spp. (27.0%); no other microorganisms (such as *Coxiella*, *Borrelia*, *Anaplasma*, *Ehlrichia*, *Rickettsia*, *Hepatozoon*, *Babesia*, and *Theileria* species) were detected. Most *Wolbachia* spp. were detected in *P. jenynsii* (22/35, 64.7%), and *Bartonella* spp. were most frequently found in *P. hindlei* (10/20, 50.0%) (Table 4).

**Table 4:** Distribution of endosymbionts detected in bat flies

Bat fly species	Bat host species (n)	*Wolbachia*	*Bartonella*
Nycteribiidae			
*Nycteribia allotopa* (2)	*Miniopterus fuliginosus* (2)	0	1
*Nycteribia parvula* (2)	*Miniopterus fuliginosus* (2)	2	0
*Nycteribia* sp.^a^ (6)	*Miniopterus fuliginosus* (2)	0	1
	*Myotis macrodactylus* (3)	0	1
	*Rhinolophus ferrumequinum* (1)	0	0
*Nycteribia* sp.^b^ (2)	*Miniopterus fuliginosus* (2)	0	0
*Penicillidia jenynsii* (26)	*Miniopterus fuliginosus* (22)	20	3
	*Myotis macrodactylus* (3)	2	0
	*Rhinolophus ferrumequinum* (1)	0	0
*Phthiridium hindlei* (15)	*Rhinolophus ferrumequinum* (15)	10	10
Unidentified (13)	*Rhinolophus ferrumequinum* (5)	1	1
	*Miniopterus fuliginosus* (8)	0	1
Subtotal (66)		35 (53.0)	18 (27.3)
Streblidae			
*Brachytarsina* sp.^c^ (8)	*Rhinolophus ferrumequinum* (8)	0	2
Subtotal (8)		0 (0.0)	2 (25.0)
Total (74)		35 (47.3)	20 (27.0)

^a^The closest GenBank matching species is *Nycteribia pleuralis*

^b^The closest GenBank matching species is not identified

^c^The closest GenBank matching species is *Brachytarsina kanoi*

### Sequence and phylogenetic analyses of bat fly-mediated pathogens and endosymbionts

*Wolbachia* spp. in *P. hindlei* share 97.7–99.8% sequence identity with *Wolbachia*-hosting spiders, moths, and beetles (GenBank: KT319069, FM883705, KC578235). *Wolbachia* spp. in *N. parvula* and *P. jenynsii* share 95.4–98.5% sequence identity with *Wolbachia* spp. isolated from cockroach and termite (GenBank: AJ292345, FJ390318, DQ457402) and 95.0–96.7% sequence identity with that isolated from nematode (GenBank: FR827926, AJ628414).

**Fig. 4:**
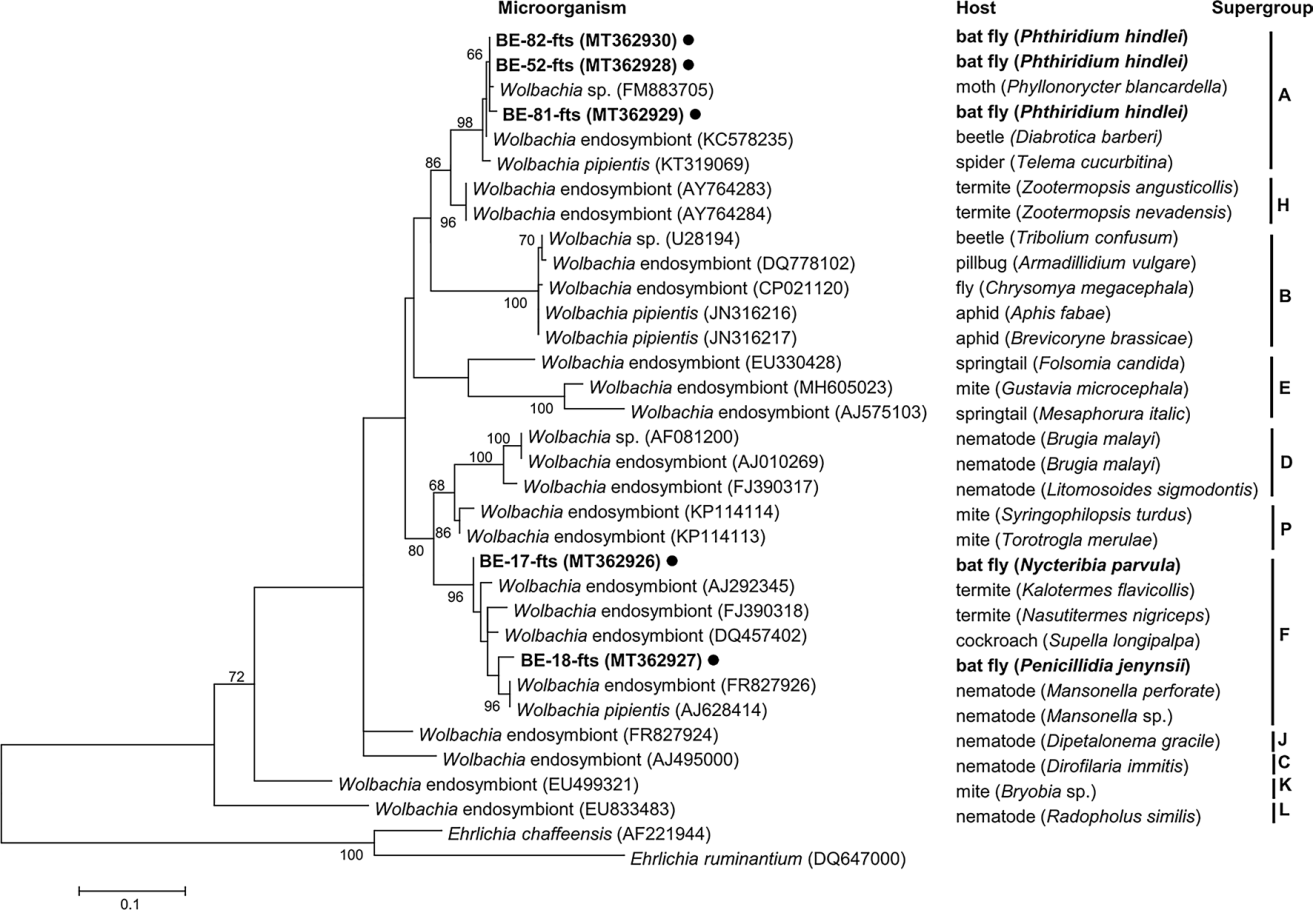
A phylogenetic tree was constructed with *Wolbachia ftsZ* gene-amplifying sequences generated in this study using the maximum likelihood method based on the Tamura-Nei model (1000 replicates). Sequences identified in this study are marked with black circles (●) with isolated ID and host species scientific name. The *Ehrlichia* sequences were used as outgroup

Through phylogenetic analysis of the *Wolbachia* spp., the three sequences from *Phthiridium* spp. bat flies were clustered as supergroup A, and the other two sequences from *Nycteribia* spp. and *Penicillidia* spp. bat flies were clustered as supergroup F (Fig. 4) [17].

**Fig. 5:**
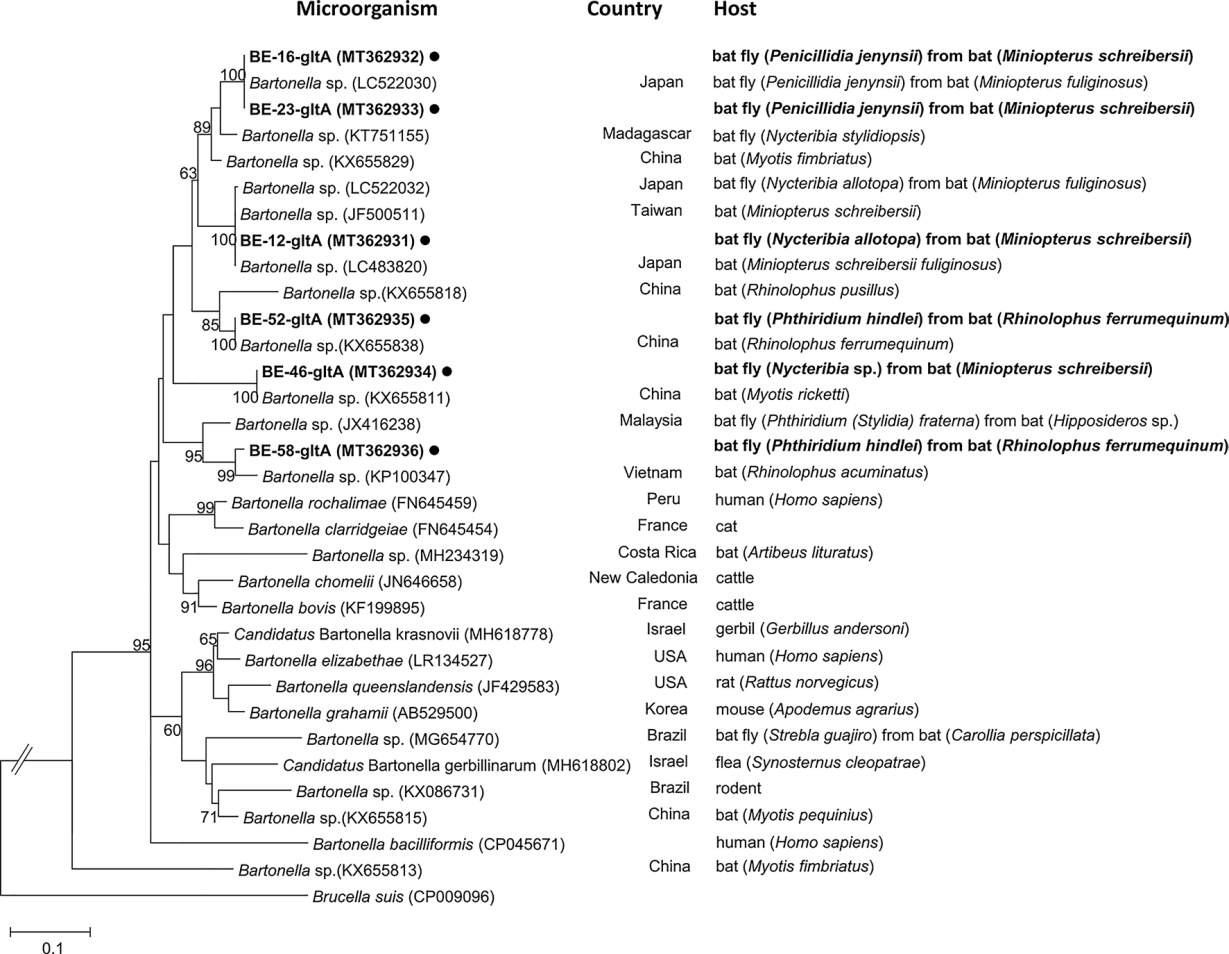
A phylogenetic tree was constructed with the *Bartonella* gltA gene-amplifying sequences generated in this study using the maximum likelihood method based on the Tamura-Nei model (1000 replicates). Sequences identified in this study are indicated by black circles (●) with isolated ID and host species scientific name. The *Brucella* sequences were used as outgroup

In the phylogenetic analysis of *Bartonella gltA* amplified from the bat flies in this study, six representative sequences were obtained from five bat fly species with 86.6–91.9% sequence identity (Fig. 5). *Bartonella* sequences from the bat fly *P. hindlei* (GenBank: MT362935) presented 91.9% identity with *P. jenynsii* (GenBank: MT362933), 91.7% with *N. allotopa* (GenBank: MT362931), and 86.6% with *Nycteribia* sp. (GenBank: MT362934). Particularly, one *Bartonella* sequence detected from the Japanese bat fly (GenBank: LC522030) had 100% sequence identities with the *Bartonella* sequences from this study (GenBank: MT362932 and MT362933). In addition, *Bartonella* sequences from the Taiwan bat (GenBank: JF500511), Japanese bat (GenBank: LC483820), and bat fly (GenBank: LC522032) had 100% sequence identity with a *Bartonella* sequence detected in this study (GenBank: MT362931).

## Discussion

According to the morphological characteristics of bats identified in Korea, 24 species have been reported from 11 genera [26, 27]. According to the key to the order Chiroptera in Korea, four bat families (Miniopteridae, Molossidae, Rhinolophidae, and Vespertilionidae) have been reported in Korea. In this study, 11 species belonging to seven genera in three families (Miniopteridae, Rhinolophidae, and Vespertilionidae) were identified. The collection places of bats were recorded for all except 31 specimens (these specimen data on tubes were erased because of leakage of ethanol during transfer), most of which were found in mines (79 specimens), caves (76 specimens), and the rest forests (12 specimens). *Rhinolophus ferrumequinum* was found in all regions except Gangwon; however, this does not necessarily mean that it does not occur in Gangwon. Only two bat specimens were collected from Gangwon. *Rhinolophus ferrumequinum* is known to be the most widely occurring bat in Korea [26].

A total of 74 bat flies were collected. One possible reason for this small number could be that the flies were collected from dead bats. Therefore, several bat flies would have left after the host bat died.

Molecular identification and phylogenies of bat flies have been widely utilized over the last decade to characterize different fly species [40, 41]. *COI*, in particular, has been proven a useful marker in the documentation of invertebrates and insects [41–43]. Previous studies on Korean bat fly were limited to morphological characteristics [25]. Moreover, traditional morphological identification methods are extremely time-consuming because bat flies are complex, extremely small, and diverse in species. Although this does not justify the lack of morphology-based identification, our results showed that a molecular approach would be useful in the quick identification of the different species of bat flies, at least at the genus level. There was a limitation that *COI* was not sufficient to reliably identify bat flies by itself at the species level because sequence data of many species are still missing from the GenBank. Therefore, in this study, *COI* sequencing enabled genus-level identification and indicated the closest GenBank match species. In some species, the most similar GenBank sequences showed a similarity of < 95% (*N. pleuralis*, 94.3%; *B. kanoi*, 93.0%), whereas others showed a similarity of > 98% (*N. allotopa*, 100%; *P. jenynsii*, 98.6%; *P. hindlei*, 99.9%). Therefore, more comparable data on *COI* will allow a clear distinction of bat flies at the species level. Previously, *COI* was also used for species identification in other families of Diptera [44, 45].

*Phthiridium hindlei* is an ectoparasite of *R. ferrumequinum*, which has not been reported in Korea previously. Most *Nycteribia* spp. were detected in *M. fuliginosus* (8/12, 66.7%), which is consistent with the findings of a previous study that collected *Nycteribia* spp. from *Miniopteru*s sp. in Korea [25].

*Penicillidia jenynsii* (family Nycteribiidae) was mostly collected from the Jeonbuk Province (24/26), with only two individuals identified in the Chungbuk Province. *Brachytarsina* sp. (family Streblidae) was confirmed only in Jeonnam, Gyeongbuk, and Ulsan Provinces, but not in Jeonbuk and Chungbuk Provinces. However, because there is a history of discovery of *Brachytarsina* sp. in Jeju Island and Gangwon Province [25] in the southernmost and northernmost regions of Korea, respectively, more population studies on ectoparasites and identification are required.

This study confirmed the high prevalence of *Wolbachia* and *Bartonella* bacteria and that *P. hindlei* was highly coinfected with *Wolbachia* and *Bartonella* spp. (9/10). According to the identification results of these pathogens and endosymbionts in this study, *Wolbachia* spp. was identified at a rate of 53.0% (35/66) in the family Nycteribiidae [*Penicillidia* sp. (22/26, 84.6%), *Nycteribia* spp. (2/12, 16.7%), *Phthiridium* sp. (10/15, 66.7%) and an unidentified Nycteribiidae species (1/13, 7.7%)] but not in the family Streblidae (*Brachytarsina* sp.). This is believed to be associated with the vertical transfer of the endosymbiont from mother to offspring through the mammary glands. Endosymbiont localization is consistently observed in all nycteribiid bat flies. In particular, *P. jenynsii* females exhibit a different pattern from that of males. In the abdominal cavity of females, larvae were found around the mammary glands, which supplied secretions and exhibited endosymbiont signals [20]. *Bartonella* spp. were identified at a rate of 27.3% (18/66) from the family Nycteribiidae [*Phthiridium* sp. (10/15, 60.7%), *Nycteribia* spp. (3/12, 25.0%), *Penicillidia* sp. (3/26, 11.5%), and an unidentified Nycteribiidae species (2/13, 15.4%)] and at a rate of 25.0% (2/8) in the family Streblidae (*Brachytarsina* sp.).

A previous study reported that the most common microparasites in bat flies were bacteria (*n* = 149), with high numbers of *Bartonella* spp. (*n* = 91, 61.0%) but few *Wolbachia* spp. (*n* = 8, 5.4%) [2]. However, in this study, *Wolbachia* spp. were detected at a higher rate (35/74, 47.3%), whereas *Bartonella* bacteria detection was less frequent (20/74, 27.0%). This could be due to the differences in sample collection time and areas.

As per the phylogeny results of *Wolbachia* and *Bartonella* bacteria, we confirmed that each species of bat flies clustered as separated roots of each type. The *Wolbachia* endosymbionts detected in our study were clustered into two supergroups, A and F. Supergroup A was found in arthropods, whereas supergroup F was found in both filariae and arthropods [17]. All supergroup A endosymbionts were detected in *P. hindlei*. Supergroup F endosymbionts were detected in other bat flies (*Penicillidia* sp. and *Nycteribia* spp.). The presence of *Wolbachia* spp. confirmed that various arthropods could be vectors, and the bat fly *Wolbachia* spp. could have sequences similar to both filariae and arthropods. In addition, the possibility of *Wolbachia* bacteria transmission through bat blood should be studied to determine the connection between bat flies and bats.

*Bartonella* endosymbionts were also grouped according to their bat fly host except *P. hindlei*. However, these sequences clustered into distinct *Bartonella* groups and were considered a separate *Bartonella* species because of its < 96% identity [46, 47]. Interestingly, in this study, with two separate branch groups, *Bartonella* host specificity in bats and bat flies was confirmed; one is *P. jenynsii* group and the other *N. allotopa* group, all of which had *Miniopterus* sp. bat hosts. In a previous study conducted in northern China, bat and *Bartonella* host specificity was recorded [48], and it was suggested that *N. allotopa* is the vector for transmitting *Bartonella* in bats [49]. However, the correlation between *Bartonella* species originating from bats and other bat fly species (another *Nycteribia* sp. and *P. hindlei*) requires further study.

*Bartonella* bacterial infection has been reported in humans and animals in African countries, where it was observed that a large number of local bat flies were positive for *Bartonella* spp. [50]. Bat ectoparasites generally exhibit high host specificity; therefore, their impact on other animal species and humans may be low, but the spread of bat-borne *Bartonella* spp. poses a global risk [29, 51]. Furthermore, it must be considered that endosymbionts of bat flies may come from their bat hosts. Recent research suggests that bat flies transfer viruses to host bats as well as humans [7]. Nycteribiids are known to host several *Bartonella* spp., and bat and *Bartonella* bacteria associations have been studied in several parts of the world, including Asia [30]. In this study, our data indicated that *Wolbachia* and *Bartonella* bacteria are associated with bat fly species. In this regard, studies on bat fly-mediated pathogens and endosymbionts are of great public health significance and require continued interest and research.

## Conclusions

This study employed morphological and molecular techniques to identify bat fly species in Korea. We also determined the distribution of *P. hindlei* and its endosymbionts. Using molecular methods, we identified several microorganisms, such as the endosymbiotic *Wolbachia* and possibly pathogenic or endosymbiotic *Bartonella* of bat flies, that are parasites for Korean bats. This is the first study to use such methods to identify Korean bat flies. Although the possibility of pathogen transmission through direct contact with a bat fly is low, subsequent studies on bat blood are required to confirm the potential for direct infection between bats and bat flies. This is an important public health concern owing to its potential for transmission to other species through bats.

## Supplementary Information

**Additional file 1: Table S1.** Primers used for species identification of bat flies and pathogen detection in bat flies.

**Additional file 2: Table S2.** PCR conditions.

## Data Availability

Data supporting the conclusions of this article are included within the article. The newly generated sequences were submitted to the GenBank database under the accession numbers: MT362937–MT362950 (*COI*:), MT362926–MT362930 (*ftsZ*), and MT362931–MT362936 (*gltA*). The datasets used and/or analyzed during the present study are available from the corresponding author upon reasonable request.

## Abbreviations

COI: Cytochrome c oxidase subunit I
ITS-1: Internal transcribed spacer I
