# Supplementary material for: Relationship among bats, parasitic bat flies, and associated pathogens in Korea
Source: Parasit Vectors. 2021 Sep 27;14:503. doi: 10.1186/s13071-021-05016-6 (PMC8477550; doi:10.1186/s13071-021-05016-6)
Supplement: Supplementary file 1 — Additional file 1: Table S1. Primers used for species identification of bat flies and pathogen detection in bat flies. [file 13071_2021_5016_MOESM1_ESM.docx]

**Additional file 1: Table S1.** Primers used for species identification of bat flies and pathogen detection in bat flies

| Species | Target gene | Primers | Sequence (5’->3’) | Product size (bp) | Reference |
| --- | --- | --- | --- | --- | --- |
| Invertebrate phyla | *COI* | LCO1491 | GGT CAA CAA ATC ATA AAG ATA TTG G | 710 | [1, 2] |
|  |  | HCO2198 | TAA ACT TCA GGG TGA CCA AAA AAT CA |  |  |
| *Anaplasma* spp. | 16S rRNA | Asp-Fc |  | 429 | Bioneer AccuPower^®^ Rickettsiales 3-Plex PCR kit |
|  |  | Asp-Rc |  |  |  |
| *Ehlrichia* spp. | 16S rRNA | Esp-F |  | 340 |  |
|  |  | Esp-Rc |  |  |  |
| *Rickettsia* spp. | 16S rRNA | Rsp-F |  | 252 |  |
|  |  | Rsp-R |  |  |  |
| *Wolbachia*  spp. | 16S rRNA | W16S-F | TTG TAG CCT GCT ATG GTA TAA CT | 890 | [3] |
|  |  | W16S-R | GAA TAG GTA TGA TTT TCA TGT |  |  |
|  | *ftsZ* | ftsZ-F | TAC TGA CTG TTG GAG TTG TAA CTA AGC CGT | 570 |  |
|  |  | ftsZ-R | TGC CAG TTG CAA GAA CAG AAA CTC TAA CTC |  |  |
| *Borrelia* spp. | 5S–23S rRNA | NC1 | CCT GTT ATC ATT CCG AAC ACA G | 390 | [4] |
|  |  | NC2 | TAC TCC ATT CGG TAA TCT TGG G |  |  |
|  |  | NC3 | TAC TGC GAG TTC GCG GGA G | 246-253 |  |
|  |  | NC4 | CCT AGG CAT TCA CCA TAG AC |  |  |
| *Hepatozoon* spp. | 18S rRNA | Hp18F | AAA CGG CTA CCA CAT NTA AGG A | 522 | [5] |
|  |  | Hp18R | AAT ACA AAT GCC CCC AAC TNT |  |  |
| *Babesia* spp*.* | 18S rRNA | Bsp-F |  | 934 | Bioneer AccuPower^®^ Babesia & Theileria PCR kit |
|  |  | Bsp-R |  |  |  |
| *Theileria* spp*.* | 18S rRNA | Tsp-F |  | 239 |  |
|  |  | Tsp-R |  |  |  |
| *Bartonella* spp. | *ITS-1* | QHVE-OF | TTC AGA TGA TGA TCC CAA GC | 736 | [6] |
|  |  | QHVE-OR | AAC ATG TCT GAA TAT ATC TTC |  |  |
|  |  | QHVE-IF | CCG GAG GGC TTG TAG CTC AG | 484 |  |
|  |  | QHVE-IR | CAC AAT TTC AAT AGA AC |  |  |
|  | *gltA* | 443f | GCT ATG TCT GCA TTC TAT CA | 790 | [7] |
|  |  | 1210r | GAT CYT CAA TCA TTT CTT TCC A |  |  |
| *Coxiella* spp. | 16S rRNA | cox16sf1 | CGT AGG AAT CTA CCT TRT AGW GG | 1321~1416 | [8] |
|  |  | cox16sr2 | GCC TAC CCG CTT CTG GTA CAA TT |  |  |
|  |  | cox16sr1 | ACT YYC CAA CAG CTA GTT CTC A | 719~813 |  |

**References**

1. Folmer O, Black M, Hoeh W, Lutz R, Vrijenhoek R. DNA primers for amplification of mitochondrial cytochrome c oxidase subunit I from diverse metazoan invertebrates. Mol Mar Biol Biotechnol. 1994;3:294-9.

2. Hornok S, Kontschan J, Kovats D, Kovacs R, Angyal D, Gorfol T, et al. Bat ticks revisited: *Ixodes ariadnae* sp. nov. and allopatric genotypes of *I. vespertilionis* in caves of Hungary. Parasit Vectors. 2014;7:202.

3. Sarwar MS, Jahan N, Shahbaz F. Molecular detection and characterization of *Wolbachia pipientis* from *Culex quinquefasciatus* collected from Lahore, Pakistan. Am J Trop Med Hyg. 2018;98:154-61.

4. Rar V, Livanova N, Tkachev S, Kaverina G, Tikunov A, Sabitova Y, et al. Detection and genetic characterization of a wide range of infectious agents in *Ixodes pavlovskyi* ticks in Western Siberia, Russia. Parasit Vectors. 2017;10:258.

5. Duplan F, Davies S, Filler S, Abdullah S, Keyte S, Newbury H, et al. *Anaplasma phagocytophilum*, *Bartonella* spp., haemoplasma species and *Hepatozoon* spp. in ticks infesting cats: a large-scale survey. Parasit Vectors. 2018;11:201.

6. Ko S, Kim SJ, Kang JG, Won S, Lee H, Shin NS, et al. Molecular detection of *Bartonella grahamii* and *B. schoenbuchensis*-related species in Korean water deer (*Hydropotes inermis argyropus*). Vector Borne Zoonotic Dis. 2013;13:415-8.

7. Billeter SA, Hayman DT, Peel AJ, Baker K, Wood JL, Cunningham A, et al. *Bartonella* species in bat flies (Diptera: Nycteribiidae) from western Africa. Parasitology. 2012;139:324-9.

8. Duron O, Jourdain E, McCoy KD. Diversity and global distribution of the *Coxiella* intracellular bacterium in seabird ticks. Ticks Tick Borne Dis. 2014;5:557-63.
