# Supplementary material for: Relationship among bats, parasitic bat flies, and associated pathogens in Korea
Source: Parasit Vectors. 2021 Sep 27;14:503. doi: 10.1186/s13071-021-05016-6 (PMC8477550; doi:10.1186/s13071-021-05016-6)
Supplement: Supplementary file 2 — Additional file 2: Table S2. PCR conditions. [file 13071_2021_5016_MOESM2_ESM.docx]

Additional file 2: Table S2. PCR conditions.

| Species (gene) | Initial denaturation | Cycles: Denaturation, Annealing, Extension | Final extension | PCR type |
| --- | --- | --- | --- | --- |
| Invertebrate phyla (*COI*) | 95°C/5 m | 35 Cycles: 95°C/30 s, 44°C/30 s, 72°C/30 s | 72°C/5 m | Single PCR |
| Rickettsiales  (16S rRNA) | 95°C/5 m | 40 Cycles: 95°C/30 s, 59°C/30 s, 72°C/30 s | 72°C/10 m | Single PCR |
| *Wolbachia* spp.  (16S rRNA) | 95C/5m | 40 Cycles: 95°C/30 s, 51°C/30 s, 72°C/30 s | 72°C/5 m | Single PCR |
| *Wolbachia* spp.  (*ftsZ)* | 95C/5m | 40 Cycles: 95°C/30 s, 55°C/30 s, 72°C/30 s | 72°C/5 m | Single PCR |
| *Borrelia* spp.  (5S–23S rRNA) | 95°C/5 m | 35 Cycles: 95°C/30 s, 60°C/30 s, 72°C/1 m | 72°C/5 m | Nested PCR |
|  | 95°C/5 m | 35 Cycles: 95°C/30 s, 59°C/30 s, 72°C/30 s | 72°C/5 m |  |
| *Hepatozoon* spp.  (18S rRNA) | 95°C/5 m | 40 Cycles: 95°C/30 s, 57°C/30 s, 72°C/30 s | 72°C/5 m | Single PCR |
| *Babesia* spp.  (18S rRNA) | 95°C/5 m | 40 Cycles: 95°C/30 s, 61°C/30 s, 72°C/30 s | 72°C/5 m | Single PCR |
| *Theileria* spp*.*  (18S rRNA) | 95°C/5 m | 40 Cycles: 95°C/30 s, 61°C/30 s, 72°C/30 s | 72°C/5 m | Single PCR |
| *Bartonella* spp.  *(ITS-1)* | 94°C/2 m | 35 Cycles: 94°C/45 s, 55°C/45 s, 72°C/45 s | 72°C/2 m | Nested PCR |
| *Bartonella* spp.  *(gltA)* | 95C/5m | 40 Cycles: 95°C/30 s, 48°C/30 s, 72°C/30 s | 72°C/5 m | Single PCR |
| *Coxiella* spp.  *(*16S rRNA) | 95°C/5 m | 35 Cycles: 95°C/30 s, 56°C/30 s, 72°C/50 s | 72°C/10 m | Semi-nested PCR |
|  | 95°C/5 m | 35 Cycles: 95°C/30 s, 56°C/30 s, 72°C/30 s | 72°C/10 m |  |
